# OP28 Evaluating Cost Effectiveness And Distributional Impact Of Breast Cancer Screening: A Distributional Cost-Effectiveness Analysis Incorporating Patient Preference

**DOI:** 10.1017/S026646232510086X

**Published:** 2025-12-29

**Authors:** Shan Jiang, Bonny Parkinson, Yuanyuan Gu

## Abstract

**Introduction:**

Hereditary breast and ovarian cancer, linked to BRCA gene mutations, poses significant health risks. Australian breast and ovarian cancer mortality rates emphasize the public health importance of effective screening. This study evaluated the cost effectiveness of population-wide BRCA screening and its equity implications, focusing on reducing health disparities across socioeconomic groups through early detection and prevention strategies.

**Methods:**

A cost-effectiveness analysis was conducted comparing population-wide BRCA screening to family-history-based testing for Australian women aged 30 to 50 years. A decision-analytic model included a decision tree and two Markov models simulating outcomes over a lifetime horizon. Transition probabilities captured risk-reducing interventions. Outcomes included quality-adjusted life years (QALYs) and costs in 2024 AUD, discounted at five percent. Socioeconomic quintiles were defined using Socio-Economic Indexes for Areas, and distributional impacts were assessed with net health benefits and equally distributed equivalent health (EDEH) using the Atkinson index. Model inputs were derived from census data and empirical studies. Opportunity costs used UK data as a proxy.

**Results:**

Screening at the age of 30 years was cost saving (incremental cost-effectiveness ratio [ICER] −AUD8,331 [−USD5,482] per QALY), while screening at ages 35 and 40 years was cost effective (ICERs: AUD28,231 [USD18,577] and AUD40,086 [USD26,384] per QALY, respectively). Screening at ages 45 and 50 years exceeded the threshold (ICERs: AUD78,666 [USD51,778] and AUD155,937 [USD102,640] per QALY). Distributional analysis showed screening at the age of 30 years increased average quality-adjusted life expectancy and EDEH, reducing health inequality between socioeconomic groups. In contrast, screening at older ages resulted in diminished benefits and exacerbated inequalities, highlighting the importance of early intervention in achieving both cost-effectiveness and equity goals.

**Conclusions:**

Population-wide BRCA screening was cost effective for Australian women at younger ages, particularly at the age of 30 years, and reduced health inequalities. Policymakers should prioritize early screening to address hereditary breast and ovarian cancer, promote health equity, and alleviate disease burden. Further strategies to enhance access among disadvantaged groups could amplify these benefits, advancing a more equitable healthcare system.

